# Minimal invasive resurfacing: an innovative technique for the superior semicircular canal dehiscence. A case series

**DOI:** 10.1093/jscr/rjac241

**Published:** 2022-05-31

**Authors:** Fabrizio Salvinelli, Francesca Bonifacio, Claudia Beccaria, Fabio Greco, Valeria Frari, Francesco Iafrati, Maurizio Trivelli

**Affiliations:** Department of Otolaringology, Fondazione Policlinico Universitario Campus Bio-Medico, Rome, Italy

## Abstract

The superior semicircular canal dehiscence is a vestibular disease recognized condition in recent years, and surgical therapy has been modeling itself over the years to ensure the control of vestibular symptoms and auditory symptoms. In this case series, the authors have experienced an intervention aimed at closing the superior semicircular canal dehiscence through the insertion of bone paté between the meninx and the residual middle cranial fossa bone wall. Seven patients underwent this intervention, they reported an improvement in all vestibular and auditory symptoms, and hearing threshold remained stable. Despite the small sample size, the difference was significant in the control of dizziness and the reduction of pulsatile tinnitus. The technique described in this article allows the control of symptoms in superior semicircular canal dehiscence, and it is a type of surgery familiar to the otosurgeon and easily replicable as it involves a modified mastoidectomy. More data are needed.

## INTRODUCTION

Minor disease was first described by Minor Loyd in 1998, and the pathology is characterized by the bony labyrinth thinning of the superior semicircular canal (SSC). During the postnatal period, the bone tissue that separates the semicircular canal from the dura mater does not seem to thicken at the SSC arched eminence level; however, only some patients show symptoms. Some studies show individuals of the same family to have this anomaly, thus assuming a genetic predisposition [1, 2]. It is hypothesized that a second event such as trauma can be the cause of the third window, triggering symptoms; this hypothesis is known as the ‘two-stroke hypothesis’, where a thinning of the structural bone is followed by a second event that creates dehiscence [3]. The symptoms are due to a reduction in the impedance of the inner ear. The increase in mechanical pressure through the area of least resistance induces ampulla stimulation of the SSC. The mechanical stimulus can be caused by nasal or glottic Valsalva with a consequent ampullofugal or ampullopetal flow, respectively, also the vibratory stimulus as low-frequency sounds creates an excitatory stimulus (Ewalds’ Second Law). Many patients with semicircular canal dehiscence syndrome (SCD) have low-frequency conductive hearing loss due to mechanical wave energy dissipation; other patients may have perceptive hearing loss due to the impedance reduction in the cochlea vestibular scale; several other patients have normal hearing.

**Figure 1:**
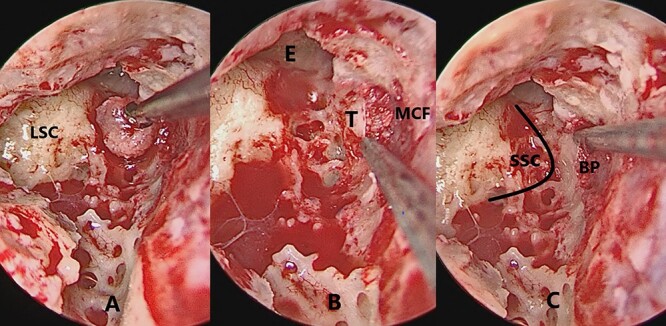
The figure shows serial images of the intervention. (**A**) Bone patè size that will be inserted into the breach. (**B**) Bone patè insertion in the gap created between the wall and the meninx of the MCF. (**C**) Distance of SSC eminence from the bone patè entry. LSC, lateral semiciruclar canal; E, eminence; T, tegmen; MCF, middle cranial fossa; SSC, superior semicircular canal; BP, bone patè.

Diagnosis is based on the medical history, audiometric examination, temporal bone CT scan and Vestibular Evoked Myogenic Potentials (Vemps). CT scan images highlight the interruption or thinning of the SSC bone labyrinth; however, several studies have highlighted a discrepancy between autopsy exams and coronal section CT images, as it seems to overestimate the defect [4].

A high-resolution ear CT scan cannot differentiate thinning from true dehiscence, as positive predictive value is reported to be 57% [5]. For these reasons, a high-resolution study with oblique plane reconstruction or a multi-slice CT with 64 helical channels is recommended, and these techniques are therefore used to reduce false positives [6]. It is known that cervical-vemps in SCD patients have a lower stimulation threshold and an increased wave amplitude, ocular-vemps have both a threshold and an increased amplitude, a burst tone is used as a stimulus at the frequency of 500 Hz; the normalization to 2000 Hz seems, however, to result in a 100% increase in sensitivity and 96% specificity compared with 52% of the standard [7]. Surgical techniques are based on closing the third window through canal plugging and resurfacing. Over the years, several surgeons have introduced innovative approaches. The materials used are bone wax, bone paté, fascia, bone dust [8].

## METHODS

In this case series, seven patients from a consecutive cohort were visited and diagnosed with Minor disease following clinical criteria established according to international studies [9]; the diagnosis was confirmed by temporal bone CT scan. The patients underwent minimal invasive resurfacing (MIR). Patients presenting a borderline clinic with other inner ear pathologies (third-window lesion, migraine, endolymphatic hydrops, enlarged Vestibular Aqueduct Syndrome [10]) were excluded from this series. The patients who did not answer to the call were excluded from the study. Patients’ demographic data are summarized in Table 1.

**Table 1:** Demographic data patients, SD (standard deviation)

Patients demographic data	Value
Total number of patients	7
Ear side (Right:left)	4:3
Average age	47.8
Sex (male:female)	3:4
Head trauma history	1 (14.28%)
Otitis history	2 (28.5%)
Bilateral disease	1 (14.28%)
Average time from the first vertigo episode (year)	6.8 (15–1)
Average follow-up (months) + SD	12$\pm$2.7

### Surgical technique: Minimal invasive resurfacing

The first surgical step is mastoidectomy, a standardized procedure that includes the identification of the lateral semicircular canal; above the SSC area the bone tegmen of the middle crania fossa (MCF) is identified and drilled with a diamond bur 0.3 cm. The dura is detached and the bone patè is inserted in a lateral and medial direction. The volume of the bone patè is enough to fill the space between the insertion area and the SSC. To reduce the risks of dural detachment, the dural dissection is minimal in SSC projection. The inside of the SSC is not violated. The muscle insertion between the meninx and the residual bone wall allows easy CSF fistula closure if it occurs (Fig. 1).

The study follow-up is 12 ± 2.7. The variables under study are monitored via questionnaire: disbalance, tinnitus, autophony, fullness and vertigo on a scale of 1/5 for preoperative and postoperative. The symptoms difference between pre and postoperative gave a statistically significant result for vertigo and pulsatile tinnitus *P* > 0.05. Six out of seven patients reported no vertigo after surgery. The size of the sample did not allow data confirmation for the rest of the symptoms taken into consideration, it is however evident for the improvement of all symptoms (Table 2). The authors have listed in on–off mode differences described by the patients on the Hennebert phenomenon, Tullio phenomenon, hyperacusis and oscillopsia (Table 3). In the audiometric exams, the authors analyzed the bone conduction pure tone average (BC PTA) for each patient at 250–500–1000–4000 Hz, whereas the air conduction pure tone average (AC PTA) was calculated for each patient at 500–1000–2000–4000 Hz. The difference between preoperative period and postoperative period was described (AAOO 1995). The hearing remained stable in six patients, in which one of the patients had already deep deafness due to a pediatric infection (Table 4). In this series, the authors did not describe intraoperative complications; no patient underwent surgical revision.

**Table 2:** Differences between preop and postop symptoms in five point intensity scale for each patient. According to Wilcoxon rank test the *P*-value is 0.031;0.25;0.125;0.063;0.031 for vertigo, autophony, disequilibrium, fullness, pulsatile tinnitus, respectively.

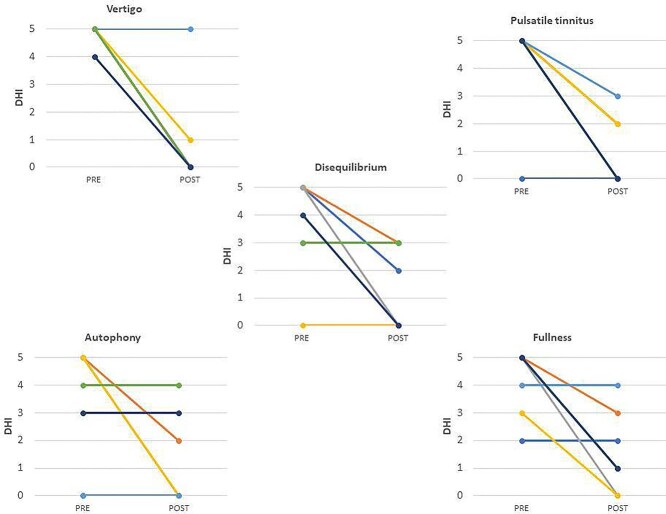

**Table 3:** For each patient, the table describes the presence [1] or absence (0) of Hennebert phenomenon, Tullio phenomenon, hyperacusis, oscillopsia in preoperative and postoperative period

	Hennebert		Tullio		Hyperacusis		Oscillopsia	
	PRE	POST	PRE	POST	PRE	POST	PRE	POST
Pz1	0	0	0	0	0	0	1	0
Pz2	1	0	1	0	1	0	1	0
Pz3	1	0	1	0	1	0	1	0
Pz4	0	0	0	0	1	0	0	0
Pz5	1	0	1	0	0	1	0	0
Pz6	0	0	1	0	1	1	1	0
Pz7	0	0	1	0	0	0	0	0

**Table 4:** In a series of seven patient, one patients shows deep hearing loss for a previous infection.

Patient	BC PTA Pre	BC PTA Post	Difference BC PTA	AC PTA Pre	PC PTA Post	Difference AC PTA
1	42	40	2	51	50	1
2	23	23	0	30	31	−1
3	40	43	−4	47	50	−3
4	53	52	1	70	70	0
5	15	15	0	20	20	0
6	15	15	0	15	15	0

In the remaining six patients, the BC PTA and AC PTA show stability in the hearing threshold compared with preoperative data.

Statistical analysis was performed with GraphPad software Inc. The Shapiro–Wilk test was used to evaluate the normality of the sample. Discontinuous variables will be described with median and IQR. The difference between preoperative and postoperative will be analyzed with Wilcoxon signed-rank test. The difference is considered to be statistically significant with *P*-values ​​ > 0.05. Biases are related to the retrospective nature of the study, different follow-up timeline for each patient and the minute sample that precludes statistical significance.

## DISCUSSION

Twenty years after the description of the pathology, several types of surgical procedures have been experimented with different approaches to the anatomical site of interest.

The approach to the middle cranial fossa allows direct exposure of the arcuate eminence; therefore it is not necessary to drill the bone above the SSC [11], and the same approach was performed using endoscopic vision and transillumination to improve dehiscence vision and reduce operating times [12]. In the transmastoid approach, the SSC is exposed with a diamond burr, the tegmen is thinned and the thin bone plate above the membranous labyrinth is removed with a curette. Subsequently, the third window closing can be done through resurfacing or canal plugging. In some cases, the dehiscence location is identified using the Buckingham mirror and closed by a structure consisting of fascia, bone patè or by cartilage. After the tegmen milling, the dura is exposed and it is gently moved from the SSC; in some cases a silastic foil is placed between the dura and the SSC during the canal resurfacing to avoid dura damaging [13, 14]. In SSC plugging, the dura is not raised from the fistula area; the labyrinth fenestration is performed near the fistula hence the canal is plugged filling the lumen with bone wax, bone patè, fascia, ect.; the dehiscence is then closed with a cartilage graft. The aim of this technique is to occlude the canal without damaging the neuroepithelium [8, 15]. The other techniques presented above expose the patient to various complications:

- Craniotomy and temporal lobe retraction in middle fossa approach: inevitably increases the hospital stay and the risk of infection [16].
- Ablation of the vestibular function: the SSC fenestration exposes the patient to the risk of sucking and injury of the membranous labyrinth. It is known that SSC skeletonization is difficult and risky due to its position; the bone labyrinth milling can cause third window syndrome worsening and chronic imbalance [17].
- Insistent milling on the tegmen: can lead to exposure of the middle cranial fossa meninges, with the risk of creating a CSF fistula or a meningocele, especially if the tegmen tends to proceed towards the SSC.
- Canal plugging: the risk of membranous labyrinth sucking and manipulating is high, resulting in vestibular hypofunction and hearing loss.

According to the literature, autophony is significantly reduced by the transmastoid canal plugging [18, 19]. The reduction of the vemps threshold can also be detected in some patients who underwent canal plugging technique [20]. A study of MRI imaging performed after canal plugging revealed a filling defect in 33% of treated patients associated with residual symptoms [21]. Therefore, it is possible to affirm that a flaw in plugging can lead to therapeutic failure. According to a 2008 meta-analysis, success rates were 97% and 93% for canal plugging and cupping respectively, but only 50% for resurfacing. Thus, comparing the techniques seems to prevail the success of plugging (underlay mode) and cupping (overlay mode). Other studies have shown a better success rate by combining these techniques. A more recent systematic review confirmed previous success rates and described the complications rates related to different techniques; 16% for resurfacing, 14% for canal plugging, 16% for the transmastoid approach, 12% for MCF approach. Among the complications described, transient and permanent perceptive hearing loss, benign paroxysmal positional vertigo, transient dizziness and facial nerve palsy have been reported [22]. Regarding audiometric exams, canal plugging seems to lead to a reduction in AC and an improvement in BC [23]. A spotlight is put to the different techniques failures due to an incorrect diagnosis. Differential diagnosis includes perilymphatic fistula, that shares the Tullio phenomenon with SCD, this pathology must be suspected in case of previous head trauma or previous ear surgery, and cannot be a result of a spontaneous phenomenon. According to the Barany Society’s Guidelines, episodic dizziness associated with fluctuating symptoms constitutes a probable Meniere disease diagnosis, these symptoms, and the conductive hearing loss is in common with SCD; in the same way, vestibular migraine shares symptoms such as aural pressure, hearing disturbances and vestibular symptoms [24].

The described technique allows the vertigo control in superior semicircular canal dehiscence; the technique does not require manipulation of the SSC, therefore the risk of membranous labyrinth injury is low. The milling does not take place at the level of the dura mater which is therefore protected. The minimal invasive resurfacing in SCD is a replicable procedure, and it resulted effective in treating disabling symptoms with a negligible risk of complications. More data are needed to confirm these initial positive data.

## CONFLICT OF INTEREST STATEMENT

The authors declare that they have no competing interests.

## FUNDING

None.
